# Moral parochialism misunderstood: a reply to Piazza and Sousa

**DOI:** 10.1098/rspb.2015.2628

**Published:** 2016-01-27

**Authors:** Daniel M. T. Fessler, Colin Holbrook, Martin Kanovsky, H. Clark Barrett, Alexander H. Bolyanatz, Matthew M. Gervais, Michael Gurven, Joseph Henrich, Geoff Kushnick, Anne C. Pisor, Stephen Stich, Christopher von Rueden, Stephen Laurence

**Affiliations:** 1 Department of Anthropology and Center for Behavior, Evolution, and Culture, University of California, Los Angeles, Los Angeles, CA 90095-1553, USA; 2 Institute of Social Anthropology, FSEV, Comenius University, 820 05 Bratislava 25, Slovakia; 3 Social Sciences Subdivision, College of DuPage, Glen Ellyn, IL 60137-6599, USA; 4 Department of Anthropology, University of California, Santa Barbara, Santa Barbara, CA 93106-3210, USA; 5 Department of Psychology and Department of Economics, University of British Columbia, Vancouver, British Columbia, Canada V6T 1Z4; 6 School of Archaeology and Anthropology, The Australian National University, Canberra, Australian Capital Territory 0200, Australia; 7 Department of Philosophy and Center for Cognitive Science, Rutgers University, New Brunswick NJ 08901-1107, USA; 8 Jepson School of Leadership Studies, University of Richmond, VA 23173, USA; 9 Department of Philosophy and Hang Seng Centre for Cognitive Studies, University of Sheffield, Sheffield S3 7QB, UK

Our paper [1] compared two competing hypotheses. The hypothesis that we label *universalistic moral evaluation* holds that a definitional feature of reasoning about moral rules is that, *ceteris paribus*, judgements of violations of rules concerning harm, rights or justice will be insensitive to spatial or temporal distance or the opinions of authority figures. The hypothesis that we label *moral parochialism,* consonant with a variety of theories of the evolutionary origins of morality, holds that, because moral judgements primarily serve to navigate local social arenas, remote events will not activate the mechanisms that generate negative moral evaluation to the same extent as events occurring in the here and now, whereas the consent of local authority figures will temper condemnation. Hence, moral parochialism predicts that the collective output of the faculties responsible for moral judgement will exhibit a reduction in the severity of judgement as a function of spatial or temporal distance or the opinions of local authority figures. We provided evidence from seven diverse societies, including five small-scale societies, showing that such reductions in severity judgements exist in all of the societies examined.

Piazza and Sousa [2] argue that our data do not support parochialism, and instead support universalism, because

(1) Only a minority of our participants *reversed their initial judgement* of the wrongness of an action (from wrong to not wrong or good) when it was subsequently framed as having occurred long ago or far away, or as having been sanctioned by authority figures.
(2) Our use of *graduated moral judgements*, rather than dichotomous judgements, is inappropriate.
(3) Only a minority of our participants *diminished the severity of their initial judgement* of the wrongness of an action when it was subsequently framed as having occurred long ago or far away, or as having been sanctioned by an important person.

These objections stem from misunderstandings of moral parochialism and the evolutionary reasoning behind it.

Moral parochialism does not hold that moral judgements should necessarily be *insensitive* to wrongdoings distant in space or time or to violations sanctioned by local authorities, but rather that the collective output of the faculties responsible for moral judgement will exhibit a reduction in the severity of judgement as a result of such factors. As we explicitly noted, ‘remote events will not activate the evolved mechanisms undergirding negative moral evaluation to the same degree as actions that occur in the here and now. This is not to say that actors should assess remote transgressions as acceptable. Rather, remote events should simply trouble actors less than immediate events, evoking weaker sentiments and eliciting less overt condemnation.’

The heart of our thesis is a cost/benefit analysis wherein the benefits of moral disapproval mainly derive from reputation enhancement and the avoidance of higher-order punishment, in addition to the cost/benefit ratios of addressing harmful actions occurring at a distance or with the consent of local authorities. Many factors will affect such cost/benefit analyses; hence, whereas the primary benefits of moral judgement accrue from judgements regarding local matters, especially those concerning one's ingroup, this does not mean that moral judgement should not function at all regarding more distant matters, merely that they should be judged to be of less importance. Thus, moral parochialism does not require that *any* participants *ever* reverse their judgements from condemnation to neutrality or praise when judgements concern matters spatially or temporally distant or sanctioned by authorities—merely that condemnation will tend to diminish. Whether or not, for a given transgression and a given participant, this diminution will reach the point of indifference is an empirical question.

Sceptical that moral judgement is graded, Piazza and Sousa argue that our five-point evaluative scale should be replaced with a dichotomous wrong/not wrong categorization, and thus that our analyses employing said scale are uninformative. While both folk intuition and formal judicial systems around the world suggest that moral judgement is indeed graded, nevertheless, without accepting Piazza and Sousa's premise, we can settle the matter by conducting additional analyses, asking whether the use of a dichotomous variable changes our findings. We therefore recoded our five-point-scale responses into two categories (‘bad’ and ‘extremely bad’ = 0, all other responses = 1). Using the R package glmer2stan, we fitted the data to a series of binomial general linear models, using model comparison via deviance information criterion weights to select the best models. Results, presented in tables S1 and S9 in the electronic supplementary material, reveal that a model encompassing all seven societies sampled clearly displays evidence of moral parochialism. Examining each society individually (see the electronic supplementary material), in only one of the seven societies is parochialism no longer supported. Hence, even after dichotomizing our response variable per Piazza and Sousa's objection—thereby substantially reducing the resolution of our data—we still observe strong evidence for moral parochialism.

Piazza and Sousa assert that moral parochialism fails because a majority of participants in our sample did not reduce their initial wrongness judgements when queried regarding spatially or temporally distant events or authority approval. Both their reasoning and their use of descriptive statistics disregard key considerations. First, for many normative reasons, participants can be expected to maintain the same response across conditions. For example, cultural proscriptions are usually not phrased in parochial terms, hence self-presentation concerns can be expected to often lead to consistency. Whatever the source of consistency, were parochialism not a substantial factor in moral judgement, then changes from the baseline judgement would not be patterned. Piazza and Sousa's table 2 thus fails to afford the crucial comparison, namely the ratio between the fraction of participants who decreased their condemnation and the fraction who increased it. As evident in electronic supplementary material, table S17, per moral parochialism, for the vast majority of such comparisons, far more participants decreased their condemnation than increased it. Averaging across the societies sampled (see electronic supplementary material, table S18), in the authority condition the percentage of participants who decreased their condemnation is more than four times as large as the percentage that increased it, whereas this percentage is more than nine times larger in the temporal condition and more than 11 times larger in the spatial condition.

For at least three methodological reasons, our study probably underestimates the extent to which people reduce the severity of their moral judgements in response to our manipulations. First, our brief vignettes (terse by design, to facilitate cross-cultural comparison), framed as hypothetical, are a far cry from real moral transgressions committed, respectively, either by known members of one's community or anonymous distant strangers. Given many sources of individual variation, only the most sensitive individuals will respond to weak stimuli. Accordingly, when using such stimuli, finding the predicted patterns in a substantial minority of participants across diverse societies constitutes evidence in support of an evolutionary explanation. Our use of brief hypothetical vignettes is thus akin to using rubber snakes to test for an evolutionarily grounded fear of ancestrally relevant threats—if a quarter of participants around the world were found to be frightened by rubber snakes but not by frayed electrical cords, it would be reasonable to conclude that fear of snakes derives from a species-typical evolved psychology.

Second, our five-point scale—employed to capture changes in degree while being accessible to participants unfamiliar with scales—may well have obscured substantial variation in moral judgement, because it only offered two grades of condemnation. A fine-grained scale may reveal that many more participants shift their moral judgements when evaluating remote transgressions or those sanctioned by authorities. Similarly, because response options were constrained, our results cannot illuminate issues of magnitude. While many factors may cause participants to alter their responses across conditions, moral parochialism predicts that the magnitude of such changes should be greater for changes that involve a reduction in condemnation relative to those that involve an increase.

Third, our dependent measures—judgements communicated to a researcher by pointing to a linear scale—were an intentionally shallow, cross-culturally replicable simulation of the sorts of community discussions that are the domain of actual moral judgement. Our dependent measures differed from real life in that they entailed few costs to participants. Cost/benefit considerations are central to moral parochialism, hence it is likely that investigations in which moral judgements have more substantive consequences (e.g. expending resources to penalize wrongdoers abroad versus locally) will enhance the degree of parochialism observed.

In sum, Piazza and Sousa (i) attribute predictions to moral parochialism that it does not entail; (ii) present descriptive statistics that mask rather than reveal key features of the patterns at issue; (iii) assert that analyses using graduated moral judgements are misleading when, in actuality, employing dichotomous judgements produces essentially the same results; and (iv) fail to appreciate methodological factors that must be taken into account when assessing research conducted across diverse societies. While Piazza and Sousa's critique thus does little to undermine the evidence for moral parochialism, nevertheless, it does constructively draw attention to variation in moral judgement. Even after exploring the methodological considerations discussed above, research is likely to reveal substantial individual differences in moral parochialism, possibly including a set of individuals who are staunchly universalist. The evolutionary and ontogenetic sources of such variation—including cultural differences, a pattern clearly evident in our results—merit investigation.

## Supplementary Material

Electronic Supplementary Materials To Accompany Fessler et al. Moral Parochialism Misunderstood: A reply to Piazza and Sousa
