# Supplementary material for: Moral parochialism misunderstood: a reply to Piazza and Sousa
Source: Proc Biol Sci. 2016 Jan 27;283(1823):20152628. doi: 10.1098/rspb.2015.2628 (PMC4795033; doi:10.1098/rspb.2015.2628)
Supplement: Electronic Supplementary Materials To Accompany Fessler et al. Moral Parochialism Misunderstood: A reply to Piazza and Sousa [file rspb20152628supp1.docx]

**Electronic Supplementary Materials**

**To Accompany**

**Fessler et al. *Moral Parochialism Misunderstood: A reply to Piazza and Sousa***

**Ethics statement:** The research reported here was approved by the respective Institutional Review Boards of the participating institutions. Informed consent was obtained prior to participation (see Preamble to Participants in ESM for DOI: 10.1098/rspb.2015.0907).

**Data accessibility:** Data reported in the paper are archived at http://www.philosophy.dept.shef.ac.uk/culture&mind/Data/MoralParochialism/MoralParochialismData.csv.

**Competing interests:** We have no competing interests.

**Authors’ contributions:** DF wrote the manuscript with input from CH, SL, HB, and the other authors. MK and CH performed the analyses with input from HB.

**Funding:** Financial support for this research was provided by a grant from the U.K.’s Arts and Humanities Research Council (for the AHRC Culture and the Mind project), the Hang Seng Centre for Cognitive Studies, University of Sheffield, and the Rutgers University Research Group on Evolution and Higher Cognition.

**Section I**

**Results of re-analysis of data, performed using the R package glmer2stan, when the original 5-point evaluative scale is replaced with a dichotomous wrong / not wrong categorization**

*Section IA*

Model comparisons using DIC (deviance information criterion) weights to select best-fit models. *Parochial* indicates that the model includes increased odds of the judgment variable being in the 1 (i.e., neither “extremely bad” nor “bad”) category following the treatments.

**Table S1**. Omnibus models with Society as Random Factor, 7 levels

| Stage | Model | Factor | |  |  |  |  |  |
| --- | --- | --- | --- | --- | --- | --- | --- | --- |
|  |  | Intercept (F) | Subject (R) | Society (R) | Scenario (R) | Parochial (F) | DIC | DIC weight |
| Baseline | 1 | yes |  |  |  |  | 4284.93 | 0% |
|  | 2 | yes | yes |  |  |  | 3004.20 | 0% |
|  | 3 | yes | yes | yes |  |  | 2997.57 | 0% |
|  | 4 | yes | yes | yes | yes |  | 2961.34 | 0% |
|  | 5* | yes | yes | yes | yes | yes | 2681.49 | 100% |

*Note.* * = best fit model. *F* = fixed factor; *R* = random factor.

**Table S2**. Models for Tsimane’ society

| Stage | Model | Factor | |  |  |  |  |
| --- | --- | --- | --- | --- | --- | --- | --- |
|  |  | Intercept  (F) | Subject  (R) | Scenario  (R) | Parochial  (F) | DIC | DIC  weight |
| Baseline | 1 | yes |  |  |  | 842.62 | 0% |
|  | 2 | yes | yes |  |  | 686.67 | 0% |
|  | 3 | yes | yes | yes |  | 682.27 | 1% |
|  | 4* | yes | yes | yes | yes | 671.40 | 99% |

*Note.* * = best fit model. *F* = fixed factor; *R* = random factor.

**Table S3**. Models for Shuar society

| Stage | Model | Factor | |  |  |  |  |
| --- | --- | --- | --- | --- | --- | --- | --- |
|  |  | Intercept  (F) | Subject (R) | Scenario (R) | Parochial (F) | DIC | DIC weight |
| Baseline | 1 | yes |  |  |  | 339.05 | 0% |
|  | 2 | yes | yes |  |  | 229.25 | 0% |
|  | 3 | yes | yes | yes |  | 221.33 | 0% |
|  | 4* | yes | yes | yes | yes | 199.32 | 100% |

*Note.* * = best fit model. *F* = fixed factor; *R* = random factor.

**Table S4**. Models for Yasawa society

| Stage | Model | Factor | |  |  |  |  |
| --- | --- | --- | --- | --- | --- | --- | --- |
|  |  | Intercept  (F) | Subject (R) | Scenario (R) | Parochial (F) | DIC | DIC weight |
| Baseline | 1 | yes |  |  |  | 1048.20 | 0% |
|  | 2 | yes | yes |  |  | 735.68 | 0% |
|  | 3 | yes | yes | yes |  | 737.09 | 0% |
|  | 4* | yes | yes | yes | yes | 698.68 | 100% |

*Note.* * = best fit model. *F* = fixed factor; *R* = random factor.

**Table S5**. Models for Karo Batak society

| Stage | Model | Factor | |  |  |  |  |
| --- | --- | --- | --- | --- | --- | --- | --- |
|  |  | Intercept  (F) | Subject (R) | Scenario (R) | Parochial (F) | DIC | DIC weight |
| Baseline | 1 | yes |  |  |  | 569.49 | 0% |
|  | 2 | yes | yes |  |  | 420.00 | 0% |
|  | 3 | yes | yes | yes |  | 408.74 | 2% |
|  | 4* | yes | yes | yes | yes | 400.72 | 98% |

*Note.* * = best fit model. *F* = fixed factor; *R* = random factor.

**Table S6**. Models for Sursurunga society

| Stage | Model | Factor | |  |  |  |  |
| --- | --- | --- | --- | --- | --- | --- | --- |
|  |  | Intercept  (F) | Subject (R) | Scenario (R) | Parochial (F) | DIC | DIC weight |
| Baseline | 1 | yes |  |  |  | 240.73 | 0% |
|  | 2 | yes | yes |  |  | 191.08 | 3% |
|  | 3* | yes | yes | yes |  | 184.38 | 86% |
|  | 4 | yes | yes | yes | yes | 188.49 | 11% |

*Note.* * = best fit model. *F* = fixed factor; *R* = random factor.

**Table S7**. Models for Storozhnitsa society

| Stage | Model | Factor | |  |  |  |  |
| --- | --- | --- | --- | --- | --- | --- | --- |
|  |  | Intercept  (F) | Subject (R) | Scenario (R) | Parochial (F) | DIC | DIC weight |
| Baseline | 1 | yes |  |  |  | 490.46 | 0% |
|  | 2 | yes | yes |  |  | 330.37 | 0% |
|  | 3 | yes | yes | yes |  | 299.80 | 0% |
|  | 4* | yes | yes | yes | yes | 263.60 | 100% |

*Note.* * = best fit model. *F* = fixed factor; *R* = random factor.

**Table S8**. Models for California society

| Stage | Model | Factor | |  |  |  |  |
| --- | --- | --- | --- | --- | --- | --- | --- |
|  |  | Intercept  (F) | Subject (R) | Scenario (R) | Parochial (F) | DIC | DIC weight |
| Baseline | 1 | yes |  |  |  | 602.71 | 0% |
|  | 2 | yes | yes |  |  | 391.46 | 0% |
|  | 3 | yes | yes | yes |  | 365.35 | 0% |
|  | 4* | yes | yes | yes | yes | 322.84 | 100% |

*Note.* * = best fit model. *F* = fixed factor; *R* = random factor.

*Section IB*

Parameters of best-fit models. Positive β values indicate increased odds of the judgment variable being in the 1 (i.e., neither “extremely bad” nor “bad”) category as a function of the treatment at issue. Effects are reported in descending order of effect size within effect type (fixed, random).

**Table S9.** Parameters of best-fit binomial logit models for an omnibus model including all seven societies sampled.

| **Parochialism Omnibus Model** |  |  |  |  |  |
| --- | --- | --- | --- | --- | --- |
| Fixed effects | Estimate | exp(β) | SE | exp CI (5%) | exp CI (95%) |
| Intercept | -5.00 | 0.007 | 0.600 | 0.003 | 0.017 |
| Temporal Treatment | 1.51 | 4.527 | 0.160 | 3.490 | 5.930 |
| Spatial Treatment | 1.49 | 4.437 | 0.160 | 3.387 | 5.812 |
| Authority Treatment | 1.09 | 2.974 | 0.170 | 2.271 | 3.935 |
| Random effects | Variance | SD |  |  |  |
| Subject | 5.290 | 2.300 |  |  |  |
| Society | 1.464 | 1.210 |  |  |  |
| Scenario | 0.203 | 0.450 |  |  |  |

**Table S10.** Parameters of best-fit binomial logit models for Tsimane’ data.

| **Parochialism model** |  |  |  |  |  |
| --- | --- | --- | --- | --- | --- |
| Fixed effects | Estimate | exp(β) | SE | exp CI (5%) | exp CI (95%) |
| Intercept | -2.50 | 0.082 | 0.440 | 0.040 | 0.162 |
| Temporal Treatment | 1.09 | 2.974 | 0.290 | 1.840 | 4.807 |
| Spatial Treatment | 0.66 | 1.935 | 0.300 | 1.185 | 3.158 |
| Authority Treatment | 0.28 | 1.323 | 0.310 | 0.803 | 2.203 |
| Random effects | Variance | SD |  |  |  |
| Subject | 2.496 | 1.580 |  |  |  |
| Scenario | 0.194 | 0.440 |  |  |  |

**Table S11.** Parameters of best-fit binomial logit models for Shuar data

| **Parochialism model** |  |  |  |  |  |
| --- | --- | --- | --- | --- | --- |
| Fixed effects | Estimate | exp(β) | SE | exp CI (5%) | exp CI (95%) |
| Intercept | -10.14 | 0 | 2.120 | 0 | 0.001 |
| Spatial Treatment | 4.30 | 73.700 | 1.360 | 11.134 | 888.917 |
| Temporal Treatment | 4.18 | 65.366 | 1.360 | 9.777 | 796.319 |
| Authority Treatment | 3.78 | 43.816 | 1.360 | 6.488 | 512.859 |
| Random effects | Variance | SD |  |  |  |
| Subject | 16.080 | 4.010 |  |  |  |
| Scenario | 1.416 | 1.190 |  |  |  |

**Table S12.** Parameters of best-fit binomial logit models for Yasawa data.

| **Parochialism model** |  |  |  |  |  |
| --- | --- | --- | --- | --- | --- |
| Fixed effects | Estimate | exp(β) | SE | exp CI (5%) | exp CI (95%) |
| Intercept | -4.83 | 0.008 | 0.530 | 0.003 | 0.019 |
| Spatial Treatment | 1.92 | 6.821 | 0.340 | 3.935 | 12.183 |
| Temporal Treatment | 1.69 | 5.420 | 0.340 | 3.096 | 9.583 |
| Authority Treatment | 1.61 | 5.003 | 0.350 | 2.858 | 8.846 |
| Random effects | Variance | SD |  |  |  |
| Subject | 6.052 | 2.460 |  |  |  |
| Scenario | 0.048 | 0.220 |  |  |  |

**Table S13.** Parameters of best-fit binomial logit models for best Karo Batak data.

| **Parochialism model** |  |  |  |  |  |
| --- | --- | --- | --- | --- | --- |
| Fixed effects | Estimate | exp(β) | SE | exp CI (5%) | exp CI (95%) |
| Intercept | -4.78 | 0.008 | 0.790 | 0.002 | 0.027 |
| Temporal Treatment | 1.20 | 3.320 | 0.420 | 1.682 | 6.619 |
| Spatial Treatment | 1.14 | 3.127 | 0.420 | 1.584 | 6.297 |
| Authority Treatment | 0.30 | 1.350 | 0.450 | 0.651 | 2.830 |
| Random effects | Variance | SD |  |  |  |
| Subject | 6.25 | 2.500 |  |  |  |
| Scenario | 0.656 | 0.810 |  |  |  |

**Table S14.** Parameters of best-fit binomial logit models for best Sursurunga data.

| **Parochialism model** |  |  |  |  |  |
| --- | --- | --- | --- | --- | --- |
| Fixed effects | Estimate | exp(β) | SE | exp CI (5%) | exp CI (95%) |
| Intercept | -5.99 | 0.003 | 1.320 | 0 | 0.014 |
| Random effects | Variance | SD |  |  |  |
| Subject | 8.585 | 2.930 |  |  |  |
| Scenario | 1.166 | 1.080 |  |  |  |

**Table S15.** Parameters of best-fit binomial logit models for best Storozhnitsa data.

| **Parochialism model** |  |  |  |  |  |
| --- | --- | --- | --- | --- | --- |
| Fixed effects | Estimate | exp(β) | SE | exp CI (5%) | exp CI (95%) |
| Intercept | -7.50 | 0.001 | 1.460 | 0 | 0.005 |
| Spatial Treatment | 2.62 | 13.736 | 0.600 | 5.366 | 38.475 |
| Temporal Treatment | 2.54 | 12.680 | 0.600 | 4.953 | 35.517 |
| Authority Treatment | 0.59 | 1.804 | 0.630 | 0.644 | 5.155 |
| Random effects | Variance | SD |  |  |  |
| Subject | 15.840 | 3.980 |  |  |  |
| Scenario | 2.56 | 1.600 |  |  |  |

**Table S16.** Parameters of best-fit binomial logit models for California data.

| **Parochialism model** |  |  |  |  |  |
| --- | --- | --- | --- | --- | --- |
| Fixed effects | Estimate | exp(β) | SE | exp CI (5%) | exp CI (95%) |
| Intercept | -8.19 | 0.001 | 1.530 | 0.000 | 0.002 |
| Authority Treatment | 3.30 | 27.113 | 0.630 | 10.278 | 79.838 |
| Spatial Treatment | 2.94 | 18.916 | 0.620 | 7.023 | 55.147 |
| Temporal Treatment | 2.70 | 14.880 | 0.620 | 5.641 | 43.380 |
| Random effects | Variance | SD |  |  |  |
| Subject | 18.923 | 4.35 |  |  |  |
| Scenario | 1.742 | 1.32 |  |  |  |

**Section II**

**Complete descriptive statistics by condition**

**Table S17**. Raw percentages of participant responses

|  | *Authority Consent* | | |  | *Temporal Distance* | | | | |  | *Spatial Distance* | | |  |  |  |
| --- | --- | --- | --- | --- | --- | --- | --- | --- | --- | --- | --- | --- | --- | --- | --- | --- |
| *Society* | *No*  *Change* | *More*  *Bad* | *Less*  *Bad* |  | *No*  *Change* | *More*  *Bad* | *Less*  *Bad* |  | *No*  *Change* | | | *More*  *Bad* | *Less*  *Bad* |  | | |
| Tsimane’ | 47.8% | 21.7% | 30.6% |  | 43.3% | 15.0% | 41.7% |  | 53.9%% | | | 12.8% | 33.3% | | |  |
| Shuar | 73.3% | 6.8% | 19.9% |  | 71.2% | 6.3% | 22.5% |  | 68.6% | | | 6.8% | 24.6% |  |  |  |
| Karo Batak | 63.8% | 6.3% | 29.9% |  | 60.7% | 5.8% | 33.5% |  | 59.8% | | | 3.1% | 37.1% |  |  |  |
| Storozhnitsa | 66.3% | 3.0% | 30.7% |  | 54.8% | 1.0% | 44.2% |  | 55.8% | | | 1.0% | 43.2% |  |  |  |
| Sursurunga | 71.2% | 11.7% | 17.1% |  | 69.3% | 11.7% | 19.0% |  | 72.7% | | | 8.8% | 18.5% |  |  |  |
| Yasawa | 52.0% | 22.8% | 25.2% |  | 59.7% | 15.4% | 24.9% |  | 56.9% | | | 15.1% | 28.0% |  |  |  |
| California | 77.5% | 2.8% | 19.7% |  | 77.5% | 3.2% | 19.3% |  | 78.0% | | | 1.4% | 20.6% | |  |  |
| Combined | 63.9% | 11.4% | 24.7% |  | 62.5% | 8.8% | 28.7% |  | 63.4% | | | 7.5% | 29.2% | |  |  |

*Note*. The percentages indicate, for each condition, the fraction of participants who i) did not change their badness ratings, ii) increased their badness ratings, or iii) decreased their badness ratings relative to the baseline condition (pooling ratings of all scenarios for each participant).

**Table S18**. Ratio of the percentage of participants who decreased their condemnation in each condition to the percentage of participants who increased their condemnation in that condition

| *Society* | *Authority Consent* |  | *Temporal Distance* |  | *Spatial Distance* |
| --- | --- | --- | --- | --- | --- |
| Tsimane’ | 1.41 |  | 2.78 |  | 2.60 |
| Shuar | 2.92 |  | 3.57 |  | 3.62 |
| Karo Batak | 4.74 |  | 5.78 |  | 11.6 |
| Storozhnitsa | 10.2 |  | 44.2 |  | 43.2 |
| Sursurunga | 1.46 |  | 1.62 |  | 2.10 |
| Yasawa | 1.11 |  | 1.62 |  | 1.85 |
| California | 7.04 |  | 6.03 |  | 14.7 |
| Average | 4.13 |  | 9.37 |  | 11.4 |
